# Size-tuned gold nanoparticles for treating lethal bacterial pneumonia

**DOI:** 10.1093/rb/rbag179

**Published:** 2026-08-18

**Authors:** Le Wang, Leni Zhong, Jie Qi, Wenfu Zheng, Xingyu Jiang

**Affiliations:** Guangdong Provincial Key Laboratory of Advanced Biomaterials, Shenzhen Key Laboratory of Smart Healthcare Engineering, Department of Biomedical Engineering, Southern University of Science and Technology, Shenzhen 518055, China; Academician Workstation, The Jiangxi Province Key Laboratory for Diagnosis, Treatment, and Rehabilitation of Cancer in Chinese Medicine, Jiangxi University of Chinese Medicine, Nanchang 330004, China; Guangdong Provincial Key Laboratory of Advanced Biomaterials, Shenzhen Key Laboratory of Smart Healthcare Engineering, Department of Biomedical Engineering, Southern University of Science and Technology, Shenzhen 518055, China; Guangdong Provincial Key Laboratory of Advanced Biomaterials, Shenzhen Key Laboratory of Smart Healthcare Engineering, Department of Biomedical Engineering, Southern University of Science and Technology, Shenzhen 518055, China; Guangdong Provincial Key Laboratory of Advanced Biomaterials, Shenzhen Key Laboratory of Smart Healthcare Engineering, Department of Biomedical Engineering, Southern University of Science and Technology, Shenzhen 518055, China; CAS Key Lab for Biological Effects of Nanomaterials and Nanosafety, National Center for NanoScience and Technology, Beijing 100190, China; School of Nanoscience and Engineering, University of Chinese Academy of Sciences, Beijing 100049, China; Guangdong Provincial Key Laboratory of Advanced Biomaterials, Shenzhen Key Laboratory of Smart Healthcare Engineering, Department of Biomedical Engineering, Southern University of Science and Technology, Shenzhen 518055, China

**Keywords:** gold nanoparticles, antibacterial activity, size-tuned, biosafety, multidrug-resistant bacterial infection

## Abstract

Targeted delivery and low toxicity are key considerations in the development of nanomaterials. Herein, we report a strategy to realize accumulation of antibacterial gold nanoparticles (GNPs) in the lungs *via* regulating particle size. We prepare two types of 3-amino-1,2,4-triazole-5-thiol-modified GNPs (A-GNPs) with distinct sizes: small A-GNPs (A-sGNPs) and large A-GNPs (A-lGNPs). The A-sGNPs preferentially accumulate in the lungs, instead of in the liver or kidneys. This targeting ability lowered their toxicity to the liver and kidneys. In contrast, the A-lGNPs exhibit the opposite distribution profile. The A-GNPs especially A-sGNPs show potent antibacterial effects against multidrug-resistant (MDR) Gram-negative bacteria. In a mouse model of pneumonia induced by MDR *Pseudomonas aeruginosa*, A-sGNPs achieve a 100% bacterial clearance rate in lung tissue and a 100% animal survival rate, demonstrating ideal therapeutic effects and low systemic toxicity in a size-dependent manner. Our work paves the way for developing nanomaterials with improved bioactivity and ameliorated side effects by tuning particle size, offering broad potential for biomedical applications.

## Graphical abstract

**Graphical Abstract:**
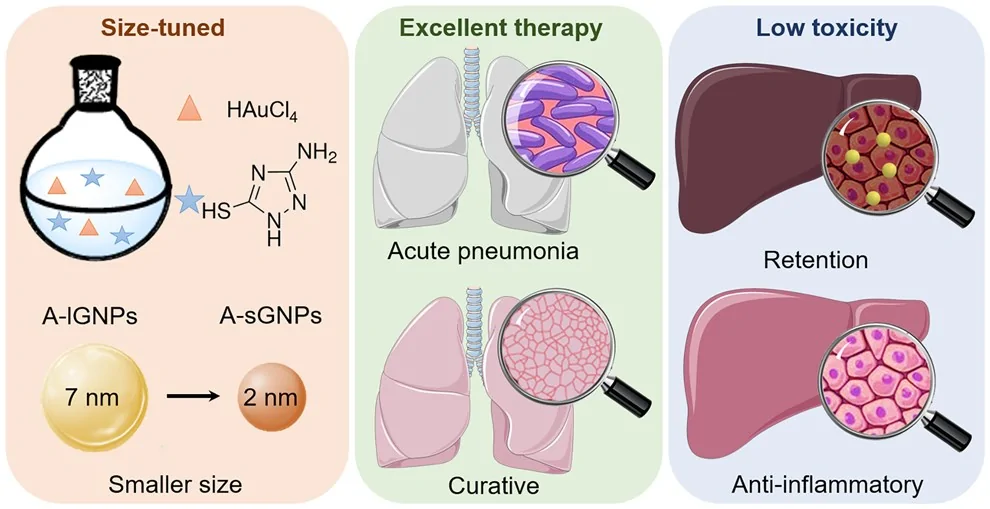

## Introduction

For many patients suffering from severe terminal illnesses, including advanced malignancies, heart failure and neurological disorders, the immediate cause of death is frequently not the primary disease but secondary pulmonary infections caused by sepsis [1]. In particular, among patients with community/hospital-acquired pneumonia admitted to the intensive care unit, mortality can reach 20–50%. Thus, the lung infection poses a huge threat to human health. Among pathogens causing lung infections, *Pseudomonas aeruginosa* (*P.a*) has emerged as a major causative agent of pneumonia, and the rise of multidrug resistance further heightens its clinical threat [2–4].

Recent progress in nanoscience has led to the development of diverse antibacterial agents, such as metal-based nanomaterials [5–7], metal oxide nanoparticles [8, 9] and antimicrobial peptides [10–12]. These materials have been extensively utilized in the treatment of multidrug-resistant (MDR) bacterial infections, demonstrating promising therapeutic efficacy. Among these materials, gold nanomaterials offer distinct advantages over other systems, including unique physicochemical properties [13, 14], facile surface functionalization and high biosafety [15–17]. These attributes have driven their increasing application in antibacterial fields, such as drug delivery [18, 19], biosensing [20–22] and nanobiotics [23–26]. However, these gold nanomaterials pose potential toxic risks to human health. For example, the toxicity of gold nanoparticles (GNPs) has been reported in recent years [27, 28]. Citrate-coated GNPs with a diameter of 14 nm showed adverse effects on human cell lines, which could easily pass through the cell membrane and accumulate into vacuoles [29]. Thirteen-nanometer PEG-coated GNPs were reported to cause acute inflammation and apoptosis in *in vivo* studies [30]. To decrease the systemic toxicity of GNPs, targeted delivery is the first consideration, which may concentrate the particles in the lesions to achieve the maximal therapeutic effects and minimal side effects. The physical and chemical properties of nanoparticles can be modulated to target specific organs and tissues. Coating nanoparticles with ligands can also modulate the biodistribution toward specific organs. For instance, galactose was used as a targeting ligand to bind to the asialoglycoprotein receptor, which is exclusively expressed on hepatocytes [31]. By modulating the internal charge of lipid nanoparticles, tissue-specific RNA delivery can be achieved in a predictable manner. Charge plays a key role in determining tissue specificity: negatively charged nanoparticles are optimal for liver and spleen delivery, whereas positively charged nanoparticles preferentially target the lungs [32]. Apart from surface charge, the particle size is another factor influencing the toxicity of antibacterial GNPs [33, 34]. Gold nanoclusters with a size less than 2 nm could be metabolized rapidly by renal clearance, and thus their toxicity could be significantly decreased *in vivo* [35, 36]. Thus, modulating the size of GNPs may decrease the toxicity and change their biodistribution *in vivo*. This strategy is applicable to systemic, local and combinatorial antibacterial regimens.

In this study, we achieve targeted therapy for bacterial pneumonia by modifying GNPs with 3-amino-1,2,4-triazole-5-thiol (ATT) to construct a gold-core/ATT-shell structure. We choose ATT as a surface ligand for its anti-oxidation effects [37]. ATT possesses amino and sulfhydryl groups. It could act as a linker: the positively charged amino group binds to negatively charged bacteria and tissues, while the sulfhydryl group conjugates with GNPs *via* Au-S bonds [38, 39]. The alveolar surface is lined with a lipid secretion layer rich in surfactants, including hydroxylated anionic phospholipids, such as phosphatidylglycerol and phosphatidylinositol [40]. The amino group of the ATT could interact with the negatively charged pulmonary surfactants, enabling the ATT-modified GNPs (A-GNPs) to achieve targeted therapy for bacterial pneumonia. Moreover, the organ distribution of the A-GNPs is size dependent. Small AGNPs (A-sGNPs) preferentially accumulate in the lungs instead of accumulating in the liver and kidneys, displaying a targeted therapeutic tendency to the lungs and a reduced toxicity to the liver and kidneys. As an application example, the A-sGNPs are used to treat MDR *P.a*-caused pneumonia, which displays potent therapeutic efficacy and low liver damage (Figure 1). Our work is insightful for designing nanomaterials with improved targeting and reduced systemic toxicity.

**Figure 1:**
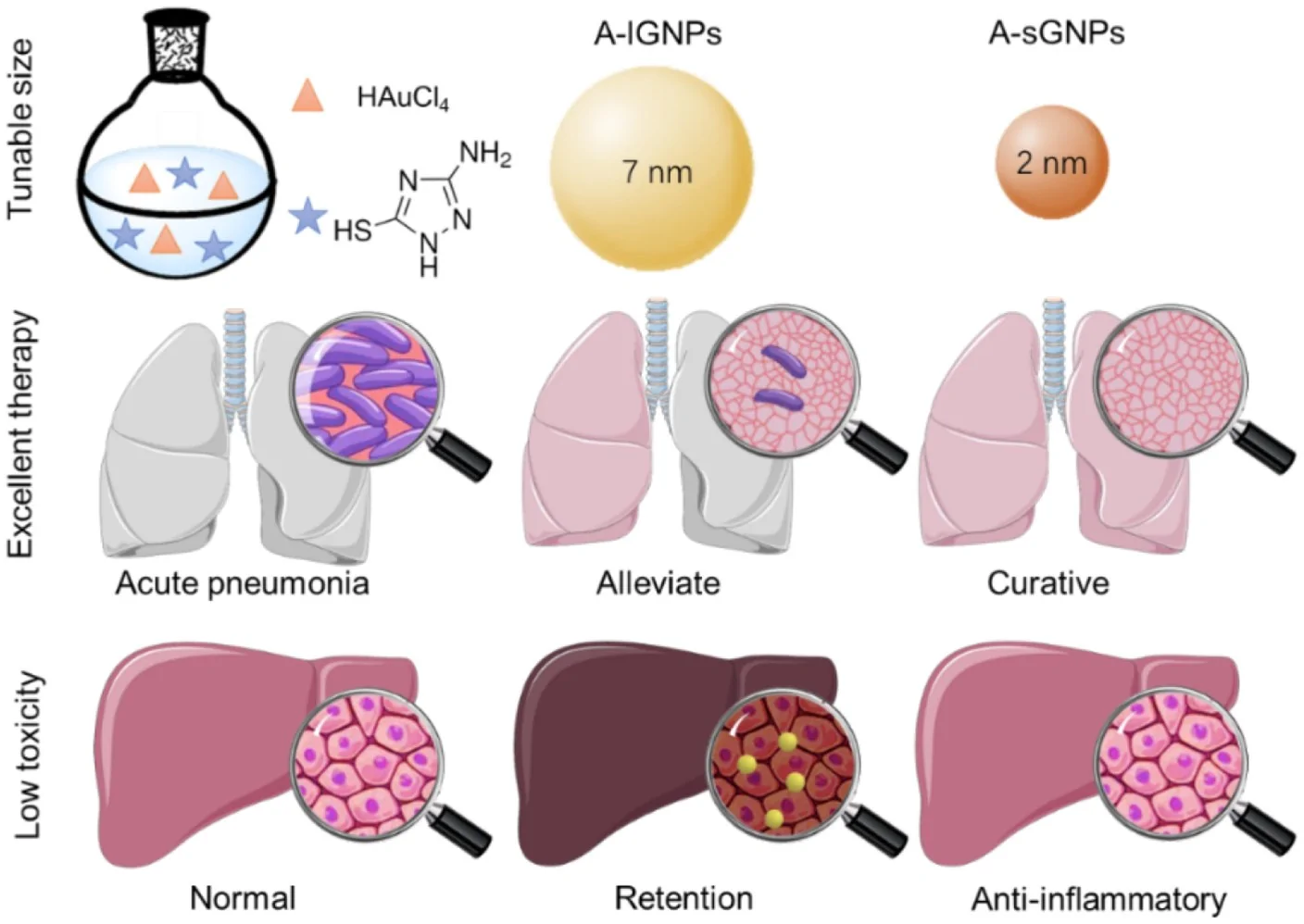
Schematic illustration of the preparation process of size-tunable and lung-targeting A-GNPs as anti-pneumonia agents without liver toxicity.

## Materials and methods

### Materials

Tetrachloroauric acid (HAuCl_4_•3H_2_O) was from Sinopharm (Sinopharm Chemical Reagent Co., Ltd.); 3-amino-1,2,4-triazole-5-thiol (MW = 116.14) was from Sigma (USA). Sodium borohydride (NaBH_4_) and triethylamine were from Aladdin (Shanghai Aladdin Biochemical Technology Co., Ltd.). All other chemicals and solvents were of reagent grade.

### Preparation and characterization of various sizes of A-GNPs

We prepared ATT-modified GNPs (A-GNPs) by the one-pot synthesis method, where the mechanism entails reduction of HAuCl_4_•3H_2_O by NaBH_4_ in water, as previously reported [25]. After mixing the ATT (0.1 mmol), HAuCl_4_•3H_2_O (0.1 mmol), triethylamine (50 μL) and Tween 80 (30 mg) in 10 mL ice water with vigorous stirring (1000 rpm) in an ice-water bath, we added the NaBH_4_ (3 mg/mL) dropwise to the above mixture for an additional 2 h. To obtain the small-sized A-GNPs, we increased the dosage of reducing agent and adjusted the pH of the reaction solution. We mixed the ATT (0.1 mmol), HAuCl_4_•3H_2_O (0.05 mmol) and triethylamine (3 mmol) in 20 mL of water and added 1 mL of NaBH_4_ (4.3 mg dissolved in 1 mL of 0.2 M triethylamine solution) with vigorous stirring (800 rpm) for 3 h at room temperature. The ATT-modified GNPs were produced by binding sulfhydryl groups to the GNPs through Au-S bonds. We collected the A-GNPs with a dialysis bag (14 kDa MWCO, Solarbio) for 24 h to remove the unreacted chemicals. We filtered the A-GNPs by a 0.22-μm filter (Millipore) and stored them at 4°C until use. We characterized the morphologies of the A-GNPs by transmission electron microscopy (TEM, Talos F200X G2, Thermo Scientific, USA). We measured dynamic light scattering (DLS) and zeta potential of the A-GNPs by a Zetasizer Nano ZS (Malvern Company, UK). We employed X-ray photoelectron spectroscopy (XPS ESCALAB 250Xi, ThermoFisher Scientific, USA) to characterize the chemical elements. We determined the UV–Vis absorption of the A-GNP solution using UV–Vis spectrophotometer (Shimadzu UV2600, Japan). We used inductively coupled plasma mass spectrometry (ICP-MS, Agilent 7700X, USA) to measure the concentrations of AGNPs.

### Bacterial strains and culture conditions

We selected Gram-negative bacteria and Gram-positive bacteria and their corresponding MDR clinical isolates, which were isolated from patients’ urine or saliva (Beijing You’an Hospital) with the patients’ consent as microbial models. Gram-negative bacteria, including *Escherichia coli* (*E. coli*, ATCC 11775), *Klebsiella pneumoniae* (*K.p*, ATCC 13883), *Pseudomonas aeruginosa* (*P.a*, ATCC 27853) and *Acinetobacter baumannii* (*A.b*, ATCC 19606). Gram-positive bacteria, including *Staphylococcus aureus* (*S.a*, ATCC 29213), *Enterococcus faecalis* (*E.f*, ATCC 29212), *Staphylococcus epidermidis* (*S.e*, ATCC 12228), *Staphylococcus haemolyticus* (*S.h*, ATCC 43253) and *Bacillus subtilis* (*B.s*, ATCC 6633), were selected in this study. MDR refers to multidrug-resistant bacteria, while CRE and VRE refer to carbapenem-resistant *E. coli* and vancomycin-resistant *E.f*, respectively. We cultured the bacteria in Luria–Bertani (LB) medium (Solarbio) for 4–6 h with shaking at 260 rpm to obtain mid-logarithmic growth-phase bacteria for the following antibacterial experiments.

### Evaluation of antibacterial activity of A-GNPs

We tested the minimal inhibitory concentration (MIC) of the AGNPs by the microdilution method. We cultured various bacteria in the LB medium and incubated them with different concentrations of A-GNPs in 96-well plates. After 24 h at 37°C, we measured the optical density of each well at 600 nm (OD_600 nm_) on the turbidity of the bacterial suspension by a microplate reader (EnSpire multimode reader, PE, USA) to obtain the antibacterial results. To further explore the interactions between bacteria and A-GNPs directly, we fixed the A-GNPs-treated bacterial samples with 2.5% glutaraldehyde and 0.1% osmic acid, cut them into superthin slices and stained the slices with 2% uranyl acetate and 0.2% lead citrate for TEM characterization (Hitachi HT7700, Japan).

We assessed antibacterial mechanisms of A-GNPs by measuring the reactive oxygen species (ROS) and ATP levels in the bacteria. We incubated the *P.a* and MDR *P.a* with different concentrations (0, 4, 64 µg/mL) of A-GNPs for 3 h at 37°C and then centrifuged the medium at 8000 rpm for 10 min to collect the supernatant. We detected the ATP levels by an ATP assay kit based on firefly luciferase (Beyotime, S0026) with a luminometer. We stained A-GNPs-treated bacteria with a ROS assay kit (Beyotime, S0033S). Specifically, we cultured the logarithmic phase bacteria with the A-GNPs for 1 h at 37°C, and treated the bacterial supernatant with 2′,7′-dichlorofluorescein diacetate (DCF-DA) for 1 h in the dark. We used a fluorospectrophotometer (Hitachi F-7100, Japan) to detect the fluorescence.

### Lung targeting of A-GNPs

We conducted the isothermal titration calorimetry (ITC) experiments using an ITC (Micro-Cal VP-ITC) instrument at 25°C. Prior to analysis, all samples were degassed and equilibrated to the experimental temperature. The titration syringe was filled with the phosphatidylinositol (PI) and phosphatidylglycerol (PG) suspension, and any air bubbles at the needle tip were carefully removed to avoid interference with the measurements; 300 µL of PG and PI solution was injected in 10 µL increments per 120 s into 2 mL of A-lGNPs and A-sGNPs, respectively. To evaluate the binding of lung surfactants to A-GNPs, the concentrations of PI and PG were fixed at 3 and 6 mM, respectively, while the concentration of A-GNPs was maintained at 0.2 mM. The data were fitted using the “One Set of Sites” model according to the shape of the binding isotherms, allowing determination of binding parameters, including the binding constants (Ka). BALB/c mice (SPF grade, female, ∼20 g, HFK Bioscience Co., Ltd., Beijing) were used to evaluate the penetration of the air-blood barrier in the alveoli of the A-GNPs. We administered A-GNPs (60 mg/kg) to mice *via* intravenous (tail vein) injection and collected bronchoalveolar lavage fluid at 2, 6 and 24 h post-injection. We exposed the trachea and inserted a puncture needle into its upper section. Subsequently, 0.4 mL of Hank’s balanced salt solution (HBSS) containing 100 µM EDTA was gently perfused through the needle. The lavage fluid was then collected by gently pressing the chest for 20 s to facilitate recovery from the thoracic cavity. Gold content in the collected samples was quantified by ICP-MS.

### Toxicology evaluation of A-GNPs

We evaluated the blood compatibility of A-GNPs by hemolysis assay. We centrifuged fresh whole blood from rats at 1500 rpm for 15 min to collect the erythrocytes. We incubated different concentrations of A-GNPs with erythrocyte suspension for 4 h at 37°C, setting saline as a positive control and water as a negative control. We collected the supernatant by centrifuging the samples at 1500 rpm for 5 min and tested the absorption by a microplate reader. We observed the morphological changes of the A-GNPs-treated cells to evaluate the biocompatibility. The cell lines used in this study were obtained from ATCC. All experiments and procedures were conducted in accordance with the relevant guidelines and regulations. We cultured human umbilical vein endothelial cells (HUVECs), mouse fibroblast (NIH-3T3) cells and Madin–Darby canine kidney (MDCK) cells in the Dulbecco’s Modified Eagle Media (DMEM) at 37°C with 5% CO_2_. After treatment with A-GNPs for different periods, we fixed the cells with a 4% formaldehyde solution (PFA) for 10 min. We stained the cells with phalloidin (5 μg/mL, Invitrogen) and Hoechst33342 (10 μg/mL, Sigma) to visualize the cytoskeleton and cell nucleus and assessed the cell density by a confocal microscopy (Zeiss LSM 710). We also analyzed the cell viability by Calcein AM/PI (Dojindo, Japan) staining. Cells were seeded into 96-well plates at a density of 3000 cells per well and treated with A-sGNPs or A-lGNPs (64 μg/mL). After 24 h of incubation, 10% CCK-8 solution (Beijing Lanjieke Technology Co., Ltd.) was added to each well, and the cells were incubated in the dark for an additional 2 h. The optical density (OD) of each well at 450 nm was then measured using a microplate reader, and cell viability was calculated accordingly. To assess the selectivity of ATT across the liver and lungs, we compared the ATT uptake capacity of human non-small cell lung cancer cell line (A549) and human hepatocellular carcinoma cell line (HepG2) at different concentrations. To further assess the overall performance of A-GNPs, we examined the cellular uptake of A-sGNPs and A-lGNPs in A549 cells.

BALB/c mice (SPF grade, female, ∼20 g, HFK Bioscience Co., Ltd., Beijing) were used to evaluate the toxicity and biodistribution of the A-GNPs. In this study, we selected intravenous administration to assess the ability of A-GNPs to efficiently circulate through the bloodstream and target the lungs. Additionally, this method is broadly applicable and clinically accepted [41, 42], while also eliminating the risk of nonspecific brain distribution that could be caused by intranasal administration. We intravenously injected A-GNPs (600 mg/kg) into mice and recorded the body weight of the mice at different time points. We collected blood samples for blood routine and serum biochemistry analysis and dissected the heart, liver, spleen, lungs, kidneys and brain for morphological observations and hematoxylin-eosin (HE) staining. We also digested the tissues and determined the gold content by ICP-MS. We determined the number of gold atoms per GNP using an established method [43]. The calculation follows the formula *N* = (*V*_GNP_ × APF)/Va, where *V*_GNP_ represents the volume of a spherical GNP, Va is the volume of a gold atom and APF is the atomic packing factor. Additionally, we calculated the surface area (*S*) and mass (*m*) of each GNP. *S* = 4π*r*², where *r* is the radius of the spherical GNP, and the mass is calculated as *m*_GNP_ ≈ *N* × *m*_Au_, *m*_Au_ ≈ *M* × 1.66 × 10^−27^kg.

### Animal model and histological analysis

We evaluated the therapeutic effect of A-GNPs *in vivo* using an acute pneumonia mouse model. All animal experiments were reviewed and approved by the Committee for Animal Research of the Southern University of Science and Technology (No. SUSTC-JY2019160). All the animal experiments complied with relevant guidelines of the Chinese government and regulations for the care and use of experimental animals. We instilled 50 μL *P.a* and MDR *P.a* (1 × 10^8^ CFU/mL) into the lungs *via* the nostril. After 1 h of infection, we divided the mice into eight groups and injected A-GNPs (5 mg/kg) into the mice through the tail vein (12 mice for each group), setting sterilized saline-treated mice as negative control and antibiotics-treated mice (cefotaxime, 2 mg/kg) as positive control. We recorded survival states and the body weight of the mice each day for three days. The mouse ear vascular network was imaged using a linear-array-based multi-scale photoacoustic/ultrasound imaging platform modified from a commercial multi-modality photoacoustic microscope (FPAM-50, PAOMTek Inc.). At different time intervals (24, 48 and 72 h), we homogenized the lungs, liver and kidneys in PBS and spread the diluted solution on LB agar plates to count the number of bacteria in the tissues. We fixed the infected lungs in 4% PFA for 24 h, embedded them in paraffin, cross-sectioned and stained them with HE for histological analysis. We collected blood samples and measured the following routine hematology parameters: white blood cells (WBC), monocytes (MON), neutrophils (NEU), lymphocytes (LYM), hemoglobin (HGB), red blood cells (RBC) and platelets (PLT). To measure the inflammatory response, we centrifuged the blood samples at 4000 rpm for 10 min to obtain the serum for blood biochemical analysis according to the manufacturer’s instructions.

## Results and discussion

### Synthesis and characterization of A-GNPs with different sizes

We synthesized the A-GNPs with different sizes by a one-step synthesis method, and the products were purified by dialysis before use. In our previous study [38], we synthesized A-GNPs primarily by adjusting the ratio of ATT and HAuCl_4_ to control the particle size. However, both large (6.75 ± 2.75 nm) and small (2.48 ± 1.64 nm) particles exhibited significant variability in size distribution (Supplementary Figure S1), leading to unpredicted toxicity. In this synthesis process, we concentrated on controlling the amount of reducing agent, synthesis pH and other parameters to obtain A-GNPs with a uniform particle size distribution across various size ranges. Transmission electron microscopy (TEM) images showed good dispersity and even size distribution of the A-GNPs with spherical shapes and average diameters of 2.18 ± 0.22 nm (A-sGNPs) and 7.33 ± 1.32 nm (A-lGNPs; Figure 2A and C). After optimizing the synthesis process, the variation in nanoparticle size distribution was reduced from over 50% to around 10%. All the A-GNPs were dark brown-colored in the solution under sunlight (Figure 2B). A-sGNPs and A-lGNPs showed an absorption peak at 520 nm, which is the typical surface plasmon resonance of GNPs (Figure 2D). Dynamic light scattering (DLS) analysis indicated that the A-sGNPs and A-lGNPs had a zeta potential of 14.76 and 10.33 mV, respectively (Figure 2E). The A-GNPs with positive charges were easy to bind with the negatively charged surfactants in the alveoli for lung targeting. The element composition of the A-GNPs was confirmed by XPS analysis. Compared with bare GNPs, the appearance of sulfur (S) and nitrogen (N) elements verified the presence of ATT on the A-GNPs (Supplementary Figure S2). Moreover, the size of A-GNPs was stable in the serum-free, DMEM or 10% FBS-containing medium over 48 h, without detectable aggregation (Supplementary Figure S3).

**Figure 2:**
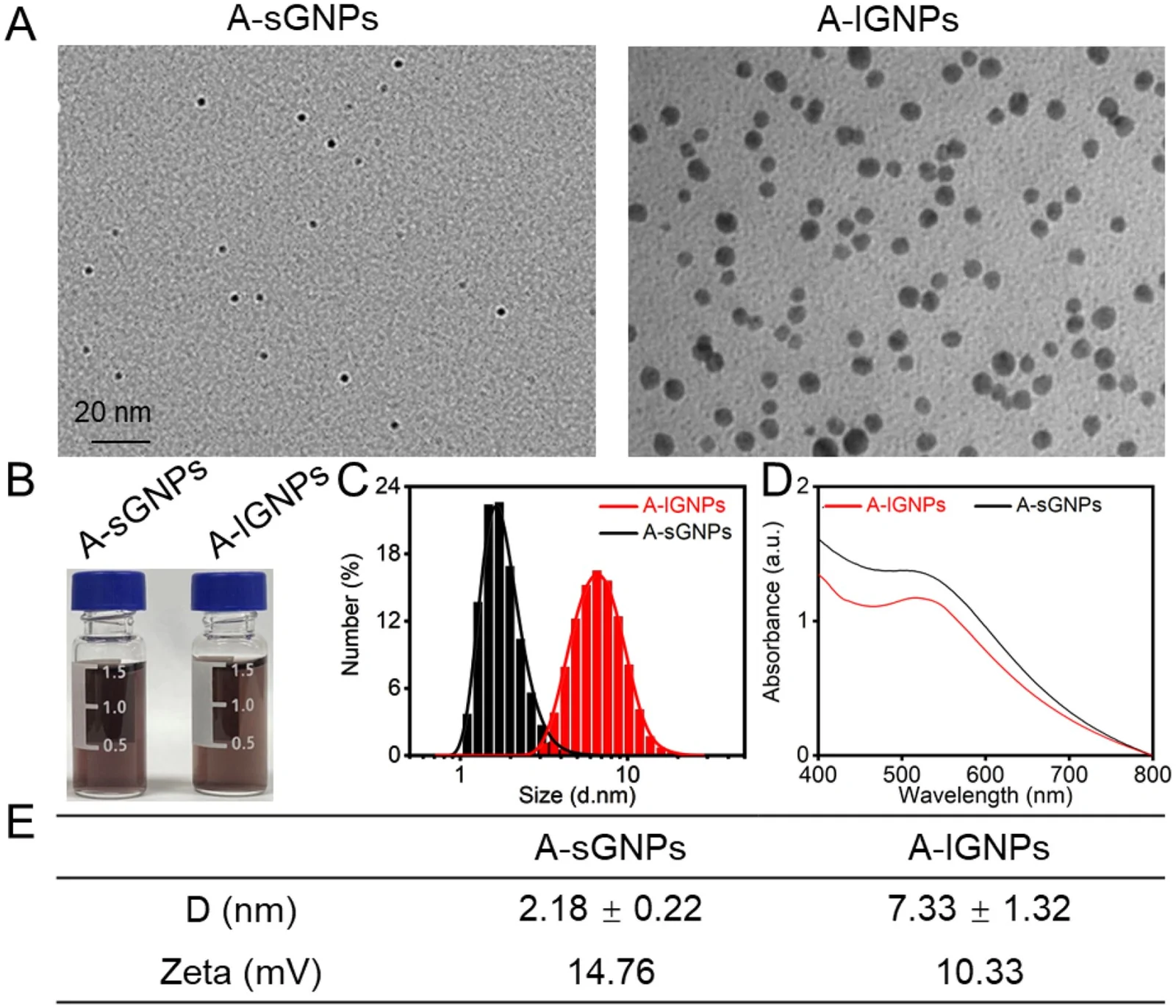
Characterization of A-GNPs with different sizes. (**A**) TEM images of A-sGNPs and A-lGNPs. Scale bar: 20 nm. (**B**) The appearance of A-sGNPs and A-lGNPs under sunlight. (**C**) Size distribution of A-sGNPs and A-lGNPs. (**D**) UV–Vis spectra of A-sGNPs and A-lGNPs. (**E**) DLS analysis of A-sGNPs and A-lGNPs.

### Antibacterial activity of A-sGNPs and A-lGNPs

We evaluated the minimum inhibitory concentration (MIC) of A-sGNPs and A-lGNPs against different species of Gram-negative (G−) bacteria and Gram-positive (G+) bacteria and their corresponding clinical MDR strains (Figure 3A). A-lGNPs had good antimicrobial activity against G- bacteria as well as the corresponding clinical strains with MIC values between 9.6 and 38.4 µg/mL. However, they exhibited no antibacterial activity against G + bacteria (MIC > 76.8 µg/mL). By comparison, A-sGNPs exhibited excellent antibacterial effects on both G- and G + bacteria (Figure 3A) and A-sGNPs showed a better antibacterial efficacy on G- bacteria than G + bacteria. Considering the highest antibacterial activity of A-GNPs in treating *P.a* and MDR *P.a*, we chose them for subsequent experiments.

**Figure 3:**
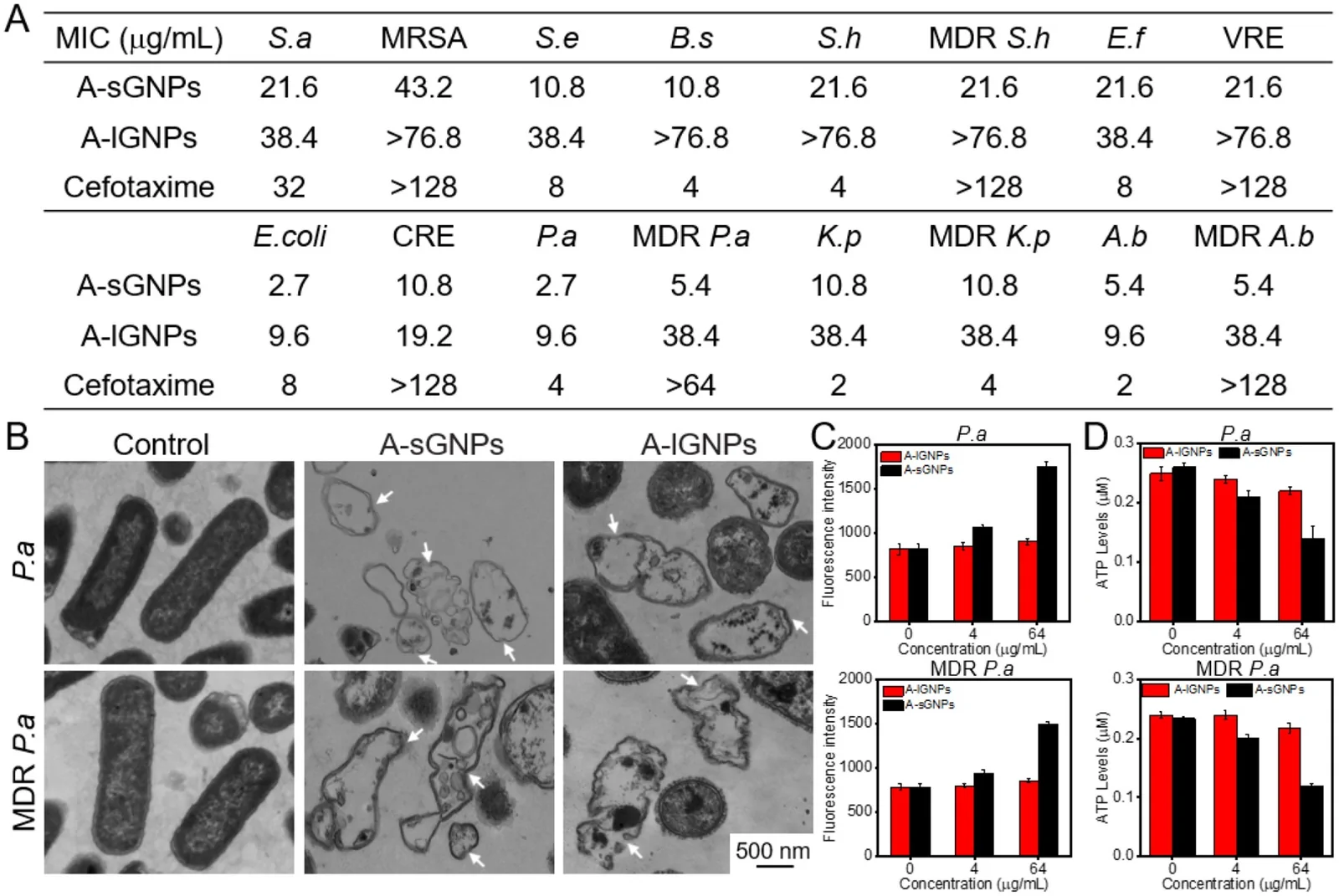
Antibacterial activity of A-sGNPs and A-lGNPs. (**A**) Antibacterial effect of A-GNPs against different bacteria. (**B**) The morphological changes of *P.a* and MDR *P.a* treated by A-sGNPs and A-lGNPs. White arrows highlight the wrinkled, blurred or severely collapsed bacterial cell walls resulting from A-GNP treatment. (**C**) The ROS levels of *P.a* and MDR *P.a* treated by A-sGNPs and A-lGNPs are measured by the total ROS assay kit. (**D**) The ATP levels of *P.a* and MDR *P.a* treated by A-sGNPs and A-lGNPs are evaluated by the ATP assay kit.

We explored the antibacterial mechanism by observing the morphological changes of bacteria under the treatment of A-GNPs using TEM. Compared with the control group, the cell wall of the A-GNPs-treated bacteria became wrinkled, blurred or even collapsed. Parts of the A-lGNP-treated and all the A-sGNP-treated bacteria lost the cytosol contents, indicating the destructive effects of the A-GNPs and better performance of the A-sGNPs in killing the bacteria. AGNPs with positive charges were easy to bind to the negatively charged bacteria, which will improve the antibacterial activities (Figure 3B). These results were consistent with the MIC results (Figure 3A). We measured the intracellular ROS levels in the A-GNPs-treated bacteria using a commercial total ROS assay kit. A-lGNPs did not induce a noticeable increased in cellular ROS, while A-sGNPs dramatically increased ROS levels in both *P.a* and MDR *P.a* (Figure 3C). We measured the intracellular ATP levels to evaluate the influence of A-GNPs on bacterial metabolism. The presence of A-sGNPs led to a decrease of ATP level in a concentration-dependent manner (Figure 3D), which may influence the survival of the bacteria. In contrast, A-lGNPs had a slight influence on the ATP-associated metabolisms (Figure 3D). The above results indicated that the antibacterial mechanisms of A-sGNPs and A-lGNPs were different. A-lGNPs inhibited the bacteria mainly by disrupting the cell wall integrity, while A-sGNPs had a multipath mechanism, including disrupting the cell wall, triggering the generation of ROS, and decreasing the ATP level.

### Biological safety and lung targeting of A-GNPs

Blood compatibility is one of the most important biosafety parameters for biomaterials. We tested the blood biocompatibility of A-GNPs by hemolysis analysis on rat erythrocytes. When the concentration of the nanoparticles rose to 128 µg/mL, A-lGNPs led to slight hemolysis, while A-sGNPs did not lead to significant hemolysis (Supplementary Figure S4), indicating the A-sGNPs have a wide range of safe concentrations for blood-contacting applications. In our previous experiments [38], we found that ATT-modified GNPs exhibited obvious cytotoxicity, with cell viability dropping below 20% at a concentration of 20 µg/mL. However, the ATT-modified GNPs showed satisfactory antibacterial activity. Therefore, we explored the possible strategy to eliminate the cytotoxicity while maintaining the antibacterial efficiency. By optimizing the synthesis method, we improved the size consistency of the A-GNPs. To assess if this strategy can reduce the cytotoxicity of A-GNPs, we further evaluated the *in vitro* cytotoxicity of the A-GNPs with different mammalian cells, including human umbilical vein endothelial cells (HUVECs), mouse fibroblast (NIH-3T3) cells and Madin-Darby canine kidney (MDCK) cells. The cell density and morphology of cells co-cultured with 64 µg/mL A-sGNPs were comparable to those of the control group, indicating favorable cell viability and proliferation. In contrast, cells treated with A-lGNPs at the same concentration showed much slower proliferation and visible cell death (Figure 4A and B and Supplementary Figure S5). Thus, by addressing the homogeneity of the nanoparticles, we greatly improved the biosafety of A-GNPs.

**Figure 4:**
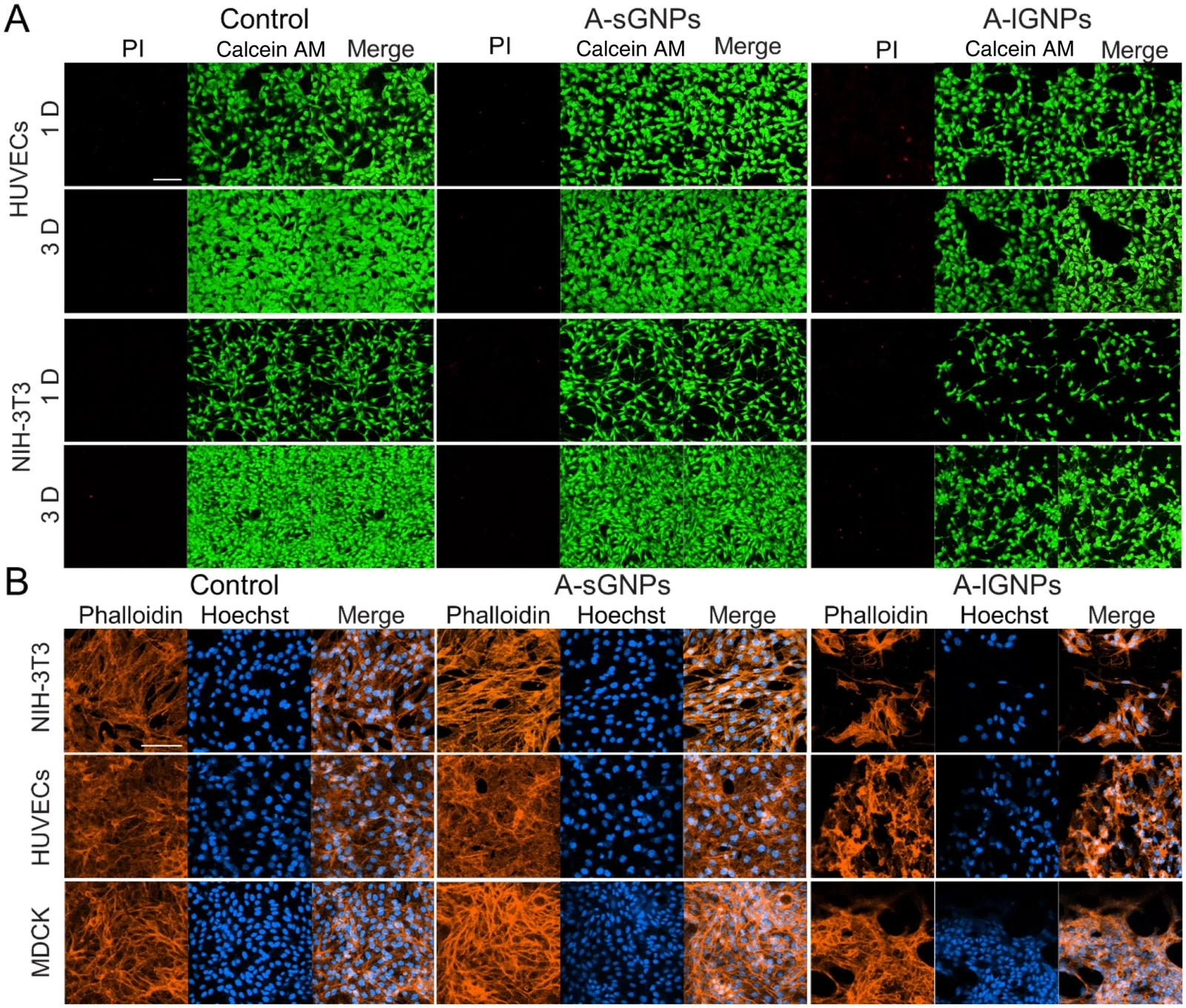
Cytotoxicity evaluation of A-sGNPs and A-lGNPs. (**A**) HUVECs and NIH-3T3 cells are incubated with A-sGNPs and A-lGNPs for 1 and 3 days. Scale bar: 100 μm. (**B**) The different cells treated with A-sGNPs and A-lGNPs are stained with rhodamine phalloidin/Hoechst to visualize the cytoskeleton and nuclei, respectively. Scale bar: 100 μm.

To assess the selectivity of ATT across the liver and lung cell lines, we compared the ATT uptake capacity of cells (human non-small cell lung cancer cell line, A549, and human hepatocellular carcinoma cell line, HepG2). At low concentrations (1 µg/mL), the ATT uptake was similar in both A549 and HepG2 cells, possibly due to that the amount of ATT is not sufficient to reach the saturation level that the cells could absorb. When the ATT concentration increased to 10 µg/mL, A549 cells internalized four times more ATT than HepG2 cells (Supplementary Table S1), suggesting that ATT possessed superior lung-targeting ability. To further assess the overall performance of A-GNPs, we examined the cellular uptake of A-sGNPs and A-lGNPs in A549 cells. A549 cells internalized 1.28-fold more A-sGNPs than A-lGNPs (Supplementary Figure S6), suggesting that the smaller size of A-sGNPs may enhance their cellular uptake and promote greater accumulation in lung cells.

We further confirmed the interaction between A-GNPs and lung surfactants through isothermal titration calorimetry (ITC) measurements. PI and PG were representative pulmonary surfactants. For PI interaction, although A-lGNPs showed a stronger calorimetric response than A-sGNPs (Supplementary Figure S7), their binding constants (Ka) to PI were comparable [(2.21 ± 0.468) × 10^4^ for A-lGNPs and (2.22 ± 0.480) × 10^4^ for A-sGNPs] (Supplementary Table S2). Compared with PI, A-GNPs exhibited a weaker Ka to PG. Similarly, the binding affinities of A-lGNPs and A-sGNPs to PG were comparable [(1.17 ± 0.185) × 10^4^ for A-lGNPs and (1.29 ± 0.219) × 10^4^ for A-sGNPs] (Supplementary Table S2). Thus, the ITC results provided direct evidence for the interaction between A-GNPs and key structural components of the lungs. Protein corona formation is a critical factor influencing the biofunctionality of nanomaterials [44, 45]. To determine if A-GNPs can form a protein corona, A-GNPs were incubated with fetal bovine serum (FBS) and mouse serum (MS) for various times, and DLS analysis was used to assess the change in particle size. The particle size and dispersion of A-GNPs showed no significant change before and after the serum incubation (Supplementary Figure S8), indicating that the effect of corona formation is negligible for A-GNPs. We also investigated the binding of A-GNPs to lung surfactants following FBS treatment to evaluate the impact of serum protein adsorption on their targeting ability. For both A-lGNPs and A-sGNPs, the heat change profiles during the interaction with PI or PG remained consistent regardless of whether the particles were treated with FBS or not (Supplementary Figure S9). Although the serum exposure resulted in a slight decrease in the binding affinity of A-GNPs to PI and a slight increase to PG, these changes were not significant (Supplementary Table S3). Thus, the presence of FBS did not significantly affect the targeting capability of A-GNPs toward lung surfactants. We further evaluated the impact of different types of serum on the antibacterial performance of A-GNPs. Both A-sGNPs and A-lGNPs maintained their antibacterial activity, ensuring the effectiveness of subsequent *in vivo* treatment (Supplementary Table S4).

We further investigated the organ targeting and toxicity of the A-GNPs by examining the nanoparticle distribution in various organs. This involved quantifying the gold content in various organs using ICP-MS. To verify the ability of A-GNPs to penetrate the air-blood barrier in the alveoli, we measured gold content in bronchoalveolar lavage fluids after the intravenous injection of A-GNPs. Regardless of particle size, A-GNPs were detected in the lavage fluid, indicating their successful translocation across the air-blood barrier (Supplementary Table S5). Notably, A-sGNPs were more efficiently retained in the lungs, leading to consistently higher gold concentrations compared to A-lGNPs at all time points. There was no difference in the lung accumulation of unmodified nanoparticles of varying sizes. In comparison to the unmodified GNPs, the distribution of A-GNPs in the lungs, regardless of size (large or small), exhibited significant improvement, indicating the lung-targeting effect of the ATT modification (Figure 5A). Human lungs have a fast blood flow rate (∼5 L/min), a low blood volume (∼75 mL) and a low blood pressure (∼6 mm Hg), which is not conducive to the retention of nanoparticles. The ATT-mediated lung tissue targeting may help the retention of A-GNPs, especially A-sGNPs, which have a significantly smaller volume (about 1/43 of that of A-lGNPs) to overcome the flow stress when they are tethered to the blood vessel wall.

**Figure 5:**
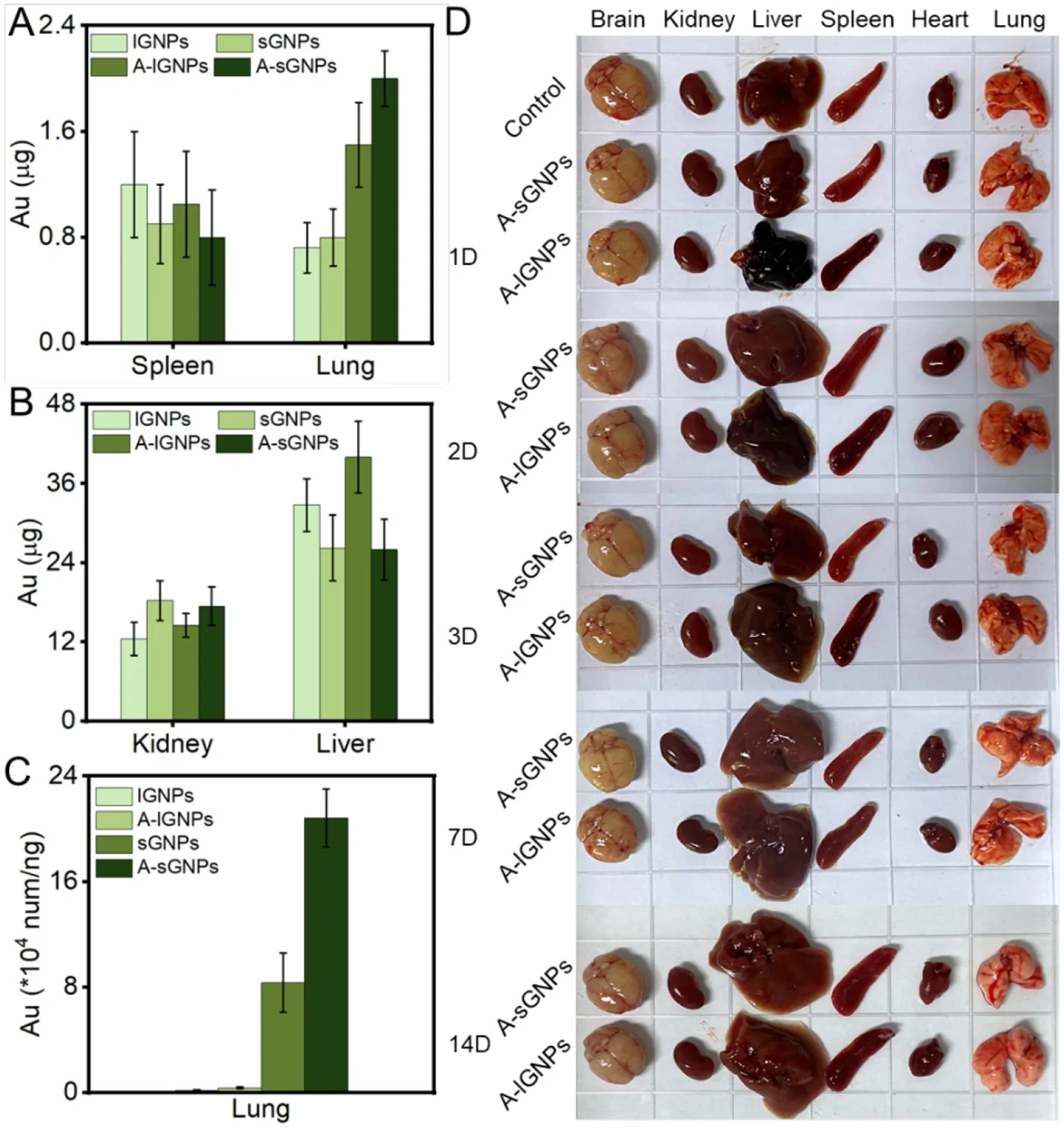
The *in vivo* distribution of different nanoparticles. (**A**) Distribution of different nanoparticles in the spleen and the lungs. (**B**) Distribution of different nanoparticles in the liver and kidneys. (**C**) Number of different nanoparticles in the lungs. (**D**) Macroscopic images of different organs after A-sGNPs and A-lGNPs treatments.

Apart from the lungs, A-lGNPs tended to accumulate in the liver, while A-sGNPs exhibited a higher tendency to distribute in the kidneys (Figure 5B). We further focused on the number of particles retained in the lungs by calculating the particle number based on the mass of a gold atom (Figure 5C). For nanoparticles of the same size, the quantity of nanoparticles with ATT modification (AGNPs) was around 2.5 times greater than those without modification (GNPs). To investigate the combined influence of particle size and surface modification on lung targeting, we analyzed the distribution of ATT-modified small particles (A-sGNPs) in comparison to the unmodified large particles (lGNPs). Notably, the lungs retained around 120 times more A-sGNPs compared to the lGNPs. Lipid nanoparticles containing a high ratio of cationic lipids could target the lungs [32]. The above results suggest a synergy between the particle surface modification and particle size in improving the lung-specific delivery and treatment.

We also performed a series of studies to evaluate the biosafety of the A-GNPs through intravenous injections (600 mg/kg) in mice. We recorded the body weight of mice at different time points with the treatment of A-GNPs. For A-sGNPs, the body weight of mice gradually increased over the treatment time, which is the same as the normal one. On the contrary, the weight of the A-lGNP-treated mice decreases at the initial stage and recovers slightly over time (Supplementary Figure S10). The major organs after the A-lGNP treatment were almost intact with normal appearance, except for the darkened color of the liver and spleen (Figure 5D). The dark brown color of the liver and spleen indicated that A-lGNPs predominantly accumulated in these two organs. This accumulation might have caused hepatic and splenic toxicity and reduce the nanoparticle concentration in the lungs (Figure 6A). Compared with the A-lGNP group, the liver and spleen of the A-sGNP group exhibited a normal color at 24 h post-administration (Figure 6A), suggesting that A-sGNPs had little tendency to accumulate in these two organs. The HE staining results verified that A-lGNPs were trapped more in the liver than the A-sGNPs (Supplementary Figure S11). On Day 3 post-administration, although the liver of the A-lGNP group recovered to the normal color, the HE staining showed an obvious change in the density of the tissue, which may be ascribed to the damage and loss of the liver cells. On the contrary, the liver of the A-sGNP group showed a normal HE staining on Day 3 post-administration. The liver of the A-lGNP group still showed an abnormal HE staining on Day 7 and gradually recovered on Day 14 (Figure 6A), indicating that the hepatotoxicity of the A-lGNPs lasted for at least 7 days. For the A-sGNP group, the liver showed a normal shape and color from the macroscopic images and the HE staining (Figure 6A), indicating an excellent biosafety.

**Figure 6:**
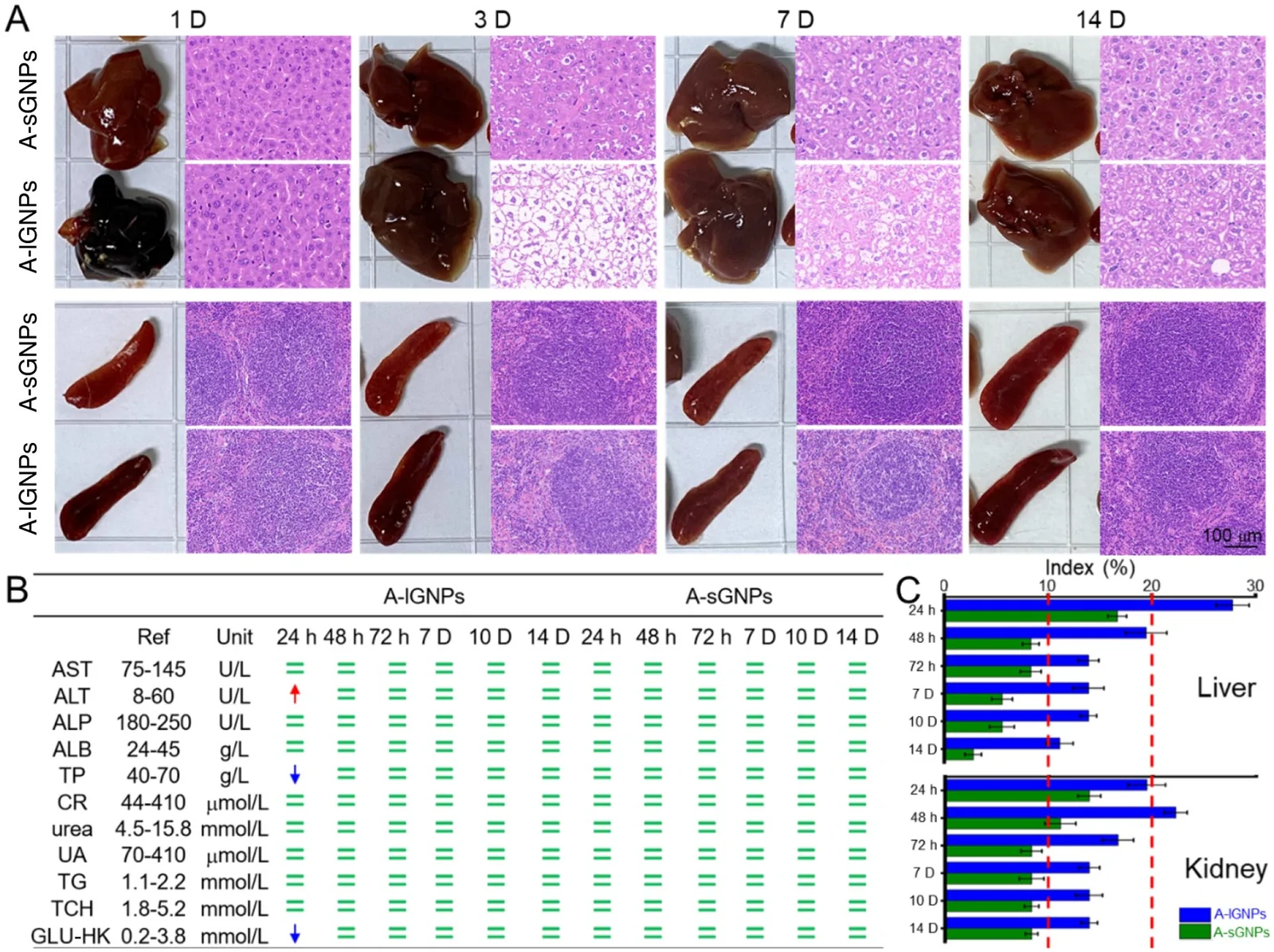
Biosafety evaluation of A-sGNPs and A-lGNPs. (**A**) Macroscopic images (from Figure 5D) and HE staining of the liver and spleen after A-sGNP and A-lGNP treatments. Scale bar: 100 µm. (**B**) Evaluation of the hepatic and renal function, immunological and inflammatory of mice at different time points treated by A-sGNPs and A-lGNPs. (**C**) The index of the hepatic and renal function of mice treated with different agents. The index of less than 10% indicates a safe level. The index higher than 20% indicates an abnormal level.

We also tested the serum levels of different biomarkers indicating the hepatic and renal functions and the immune reactions after the A-GNPs treatments. The serum levels of biomarkers in mice treated with A-sGNPs showed only minor changes (no more than 10%) after 48 h, suggesting no significant impact on the hepatic and renal functions (Figure 6B and C). However, A-lGNPs resulted in noticeable fluctuations in biomarker levels, with some levels exceeding the physiological range, indicating systemic adverse effects caused by A-lGNPs. For the blood routine test, no significant fluctuations were observed following A-GNPs treatment (Supplementary Figure S12). The above results indicated that the biocompatibility of A-sGNPs was significantly better than that of A-lGNPs.

### Antibacterial evaluation on lung infection model

We evaluate the therapeutic effect of A-GNPs *in vivo* by the nasal dropping method of *P.a* infection model on mice. *P.a* was a main pathogen of hospital-acquired infections, especially in respiratory infections and ventilator-associated pneumonia [46, 47]. We analyzed the survival rate and recorded the animal weight after the A-GNPs treatments. Compared to the control group, all mice treated with A-sGNPs (5 mg/kg) survived 72 h after *P.a* infection, demonstrating a therapeutic efficacy comparable to that of traditional antibiotics (Figure 7A). By contrast, only 75% of the mice treated with the same concentration of A-lGNPs survived (Figure 7A). For MDR *P.a* infection, the mice treated with A-sGNPs exhibited the highest survival rate (100%) among all the treatment groups, whereas none of the antibiotic-treated mice survived after 72 h, and only 60% of A-lGNP-treated mice survive after 72 h (Figure 7A).

**Figure 7:**
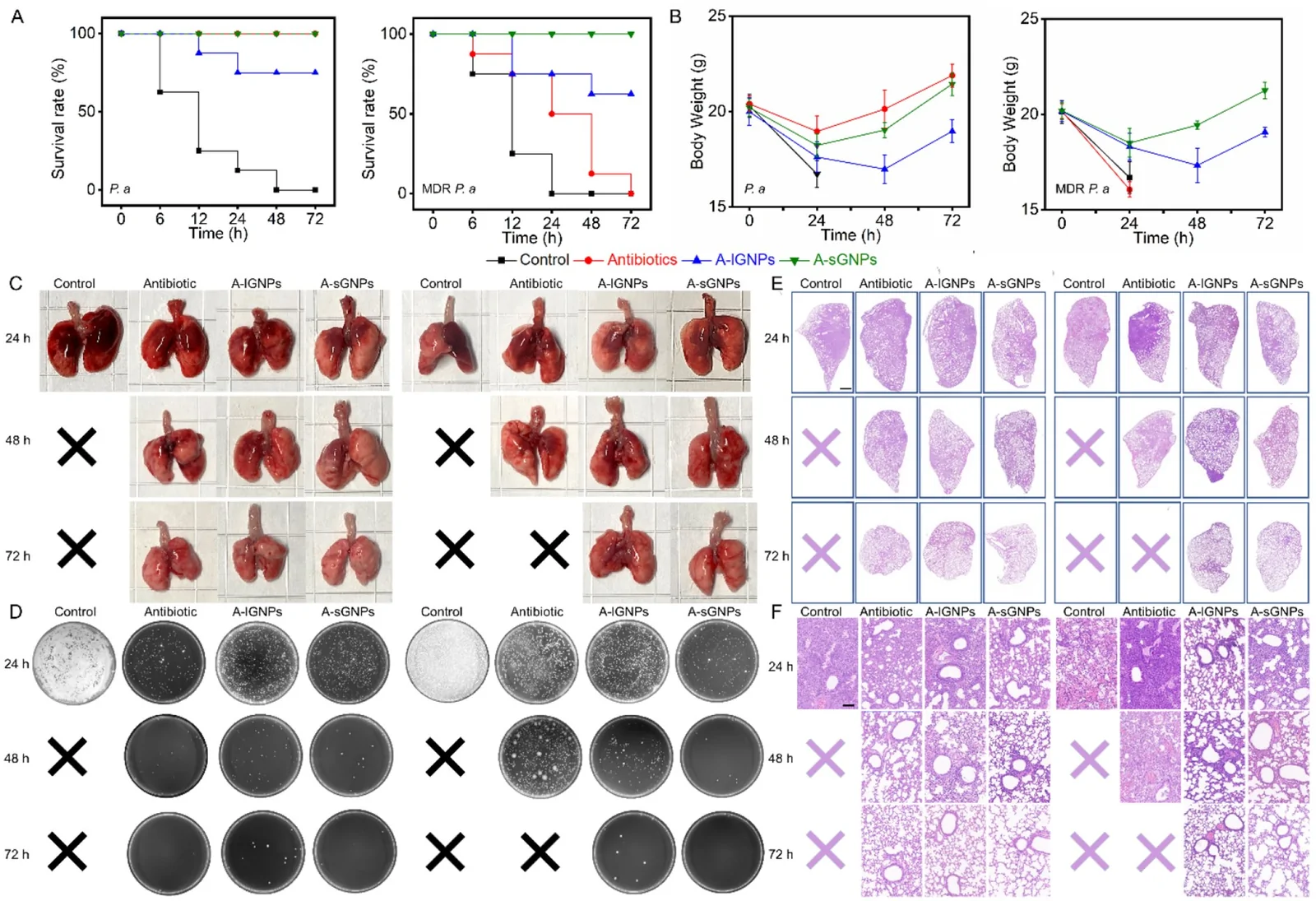
*In vivo* evaluation of the therapeutic effects of A-sGNPs and A-lGNPs on acute pneumonia. (**A**) Survival ratios of the *P.a*- or MDR *P.a*-infected mice treated by A-sGNPs, A-lGNPs, antibiotics or saline are calculated and analyzed. (**B**) Body weight of the *P.a*- or MDR *P.a*-infected mice treated by A-sGNPs, A-lGNPs, antibiotics or saline is plotted, respectively. (**C**) Photographs of representative lungs harvested from A-sGNPs, A-lGNPs, antibiotics or saline at different time points. (**D**) Bacteria in the lungs of mice with an acute pneumonia of *P.a* (left) or MDR *P.a* (right) treated by A-sGNPs, A-lGNPs, antibiotics or saline at different time points. (**E**) HE staining of histological sections of the lungs of mice with an acute pneumonia of *P.a* (left) or MDR *P.a* (right) treated by A-sGNPs, A-lGNPs, antibiotics or saline at different time points. Scale bar: 500 μm. (**F**) Enlarge part of (E). × represents the dead mice. Scale bar, 100 μm.

It is important to highlight the weight change of mice during infection treatment. During the treatment of *P.a* infection, the body weight of mice in the control and A-lGNP groups decreased sharply, while the A-sGNP and antibiotic groups maintained the normal weight at the same time points (Figure 7B). For the MDR *P.a* infection, we observed a dramatic decrease in body weight when treated with antibiotics, indicating the limited therapeutic efficacy of the antibiotics. The nanoparticles of different sizes (A-sGNPs and A-lGNPs) demonstrated therapeutic effects on infected mice. However, the weight difference of mice after 72 h post-treatment suggests that A-lGNPs may have implications for prognosis and recovery, potentially causing damage to organs such as the liver. On the other hand, treatment with A-sGNPs, which was characterized by low toxicity and high efficiency, did not affect the growth of mice at all (Figure 7B). Meanwhile, we harvested the organs and quantified the bacteria in the tissues at different time points (24, 48 and 72 h). The lungs in the control group became dark, which clearly confirms a rapid progression of pneumonia leading to death (Figure 7C). We calculated the area of acute bleeding after lung infection (Supplementary Figure S13). For the *P.a* infection, both A-sGNPs and A-lGNPs demonstrated faster efficiency in the lungs compared to antibiotics at 24 and 48 h post-treatment. This observation may be attributed to better organ targeting in the body, resulting in a greater retention effect. After successful treatment of the infection at 72 h, A-sGNPs continued to exhibit the same effect as antibiotics, whereas the A-lGNP group still showed obvious lung edema and bleeding. For the MDR *P.a* infection, the bleeding observed in the antibiotic group was alleviated but not fully recovered after treatment, leading to the death of the mice. By contrast, both A-sGNPs and A-lGNPs achieved therapeutic effects of varying degrees. A-sGNPs showed a tendency toward complete repair of the infected site, while A-lGNPs only showed a relief from bleeding. After 72 h, the bleeding area of the A-lGNPs group remained above 5%, which is up to 3.5 times that of A-sGNPs group (Supplementary Figure S13). These results may be attributed to the fact that A-sGNPs exhibited higher antibacterial efficacy than A-lGNPs. Furthermore, the tendency of A-lGNPs to accumulate in the liver restricts the number of particles capable of reaching the infected site, consequently leading to less favorable treatment outcomes and prognosis. Although most of the infected mice in the A-lGNP group survived, the bacterial number in the lung tissues revealed that the pneumonia was still serious after 72 h of treatment (Figure 7D), while the mice treated with A-sGNPs showed significant inhibition of pneumonia infection, demonstrating the prominent antibacterial effect of the A-sGNPs. Quantitative data by colony forming units (CFU) showed that the number of the residual bacteria in the A-sGNP-treated lungs was significantly lower than that in the antibiotic-treated lungs at 48 h after MDR *P.a* infection (Supplementary Figure S14), displaying better bactericidal activity of the A-sGNPs (Figure 7D). The bacteria counts in the harvested liver and kidney samples decreased significantly in the A-sGNP group (Supplementary Figures S15 and S16), indicating that bacterial infection caused mild damage to these organs. We evaluated the retention of different nanoparticles in the lung infection model after injection *via* the mice tail vein (5 mg/kg). Compared to the A-lGNPs, the clearance rate of A-sGNPs from the bronchoalveolar lavage fluid was slower (Supplementary Figure S17).

A-GNPs in infection models interacted with bacteria primarily through electrostatic interactions. Owing to their higher surface charge and stronger antibacterial activity, A-sGNPs could bind directly to bacteria, allowing them to be retained for longer periods and exert enhanced antibacterial effects compared with the nanoparticles that rely only on passive retention. In addition, infection-associated impairment of the vascular barrier and macrophage clearance in the lungs further prolongs nanoparticle retention within infected tissues, thereby extending local antimicrobial activity. Consequently, the clearance of A-sGNPs from bronchoalveolar lavage fluid was slowed (Supplementary Figure S17), indicating effective retention of A-sGNPs in the lungs and supporting their sustained antibacterial effects. Additionally, we observed the infection in lung tissue by HE staining. Obvious alveoli damage and serious pulmonary fibrosis, and neutrophils and macrophages infiltrating in a large area appear in the control group. The antibiotics exhibited partial inhibition of alveolar fusion and lung fibrosis, with obvious infiltration of neutrophils. In contrast, the A-sGNP-treated lungs maintained relatively intact alveolar structure and clear alveolar spaces, both of which are crucial for efficient gas exchange (Figure 7E and F). Furthermore, we tested the blood routine and serum biochemistry of different treatment groups. In the first 12 h, procalcitonin (PCT), serum amyloid A (SAA) and C-reactive protein (CRP), three biomarkers of inflammatory reaction, rose sharply in all groups, indicating a serious inflammatory response in the lungs. At 48 h, the levels of the inflammatory markers in the A-sGNP group were lower than those in the antibiotics group (Supplementary Table S6), revealing that the A-sGNPs could effectively alleviate the inflammation. At 72 h, the levels of all inflammatory factors in the A-sGNP group dropped to the normal range, indicating their significant anti-inflammatory effect. We standardized the serum markers of hepatic and renal function and derived a quantitative parameter that can assess alterations in hepatic and renal functions during infection treatment. A-lGNPs induced certain extent of impairment on the hepatic and renal functions (Supplementary Figure S18A), which may account for the body weight decrease. In comparison, A-sGNPs exerted relatively mild effects on hepatic function, with all variations below 10% across different time points. Conversely, the impact of A-lGNPs on the liver function was more pronounced, consistently reaching approximately twice that of A-sGNPs at 48 h and even more than 6-fold at 72 h post-administration (Supplementary Figure S19). Similar results were obtained for renal function-related indicators. The A-sGNP-treated mice showed less production of inflammation-relevant cells (WBC) and higher recovery of PLT, validating their effective antibacterial capability (Supplementary Figure S18B). These results demonstrated the outstanding antibacterial capability of A-sGNPs, which could effectively alleviate inflammation symptoms and promote the recovery of lung function *in vivo*.

## Conclusion

In this study, by modulating particle size, we achieved an improved lung retention of A-GNPs. In addition, the reduced particle size markedly lowered the accumulation of the A-GNPs in the liver, resulting in low hepatic toxicity. *In vivo* experiments demonstrated a maximal antibacterial efficacy against MDR *P.a*-caused pneumonia and a minimal systemic toxicity. Thus, modulating particle size is an effective strategy to improve therapeutic efficacy and reduce side effects of functional GNPs. We believe that this strategy will be beneficial for developing high-biosafety nanomaterials for targeted therapy. Moreover, this strategy can be extended to bacterial infections associated with inhalation therapy, implantable materials and interventional medical devices, where infections are complex and persistent.

## Supplementary Material

rbag179_Supplementary_Data
