# Supplementary material for: Size-tuned gold nanoparticles for treating lethal bacterial pneumonia
Source: Regen Biomater. 2026 Aug 18;13:rbag179. doi: 10.1093/rb/rbag179 (PMC13690419; doi:10.1093/rb/rbag179)
Supplement: Supplementary file 1 [file rbag179_supplementary_data.docx]

**Supporting Information**

# Size-Tuned Gold Nanoparticles for Treating Lethal Bacterial Pneumonia

Le Wang^1,2^, Leni Zhong^1^, Jie Qi^1^, Wenfu Zheng^1,3,4^, Xingyu Jiang^1*^

^1^ Guangdong Provincial Key Laboratory of Advanced Biomaterials, Shenzhen Key Laboratory of Smart Healthcare Engineering, Department of Biomedical Engineering, Southern University of Science and Technology, Shenzhen, Guangdong, 518055

^2^ Academician Workstation, The Jiangxi Province Key Laboratory for Diagnosis, Treatment, and Rehabilitation of Cancer in Chinese Medicine, Jiangxi University of Chinese Medicine, Nanchang, Jiangxi, 330004

^3^ CAS Key Lab for Biological Effects of Nanomaterials and Nanosafety, National Center for NanoScience and Technology, Beijing, 100190

^4^ School of Nanoscience and Engineering, University of Chinese Academy of Sciences, Beijing, 100049.

*Corresponding Author(s): Xingyu Jiang, jiang@sustech.edu.cn

Figure S1. Optimizing the synthesis method to enhance the size consistency of A-GNPs with different size ranges. ATT:HAuCl_4_ (1:1) and (10:1) from Ref. 38. A-sGNPs and A-lGNPs from figure 2A.

Figure S2. XPS spectra for the chemical element of gold (Au), sulfur (S), carbon (C), nitrogen (N) and oxygen (O) respectively on the surface of A-GNPs and bare GNPs.

Figure S3. Size distribution of the A-sGNPs and A-lGNPs in different solutions.

Figure S4. Blood compatibility of A-sGNPs and A-lGNPs. Fresh rat erythrocytes are incubated with different concentrations of (A) A-sGNPs and (B) A-lGNPs. Saline is used as a negative control and water as a positive control.

Figure S5. Evaluation of cytotoxicity by CCK-8 assay in NIH-3T3 cells and HUVECs following treatment with A-sGNPs and A-lGNPs, respectively.

Figure S6. Cellular uptake of A-sGNPs and A-lGNPs in A549 cells, quantified by measuring intracellular gold content.

Figure S7. ITC analysis of the interaction between A-GNPs and the lung surfactants. Curves correspond to the interactions of PI with (A) A-lGNPs and (B) A-sGNPs. Curves correspond to the interactions of PG with (C) A-lGNPs and (D) A-sGNPs.

Figure S8. Size distribution of the A-sGNPs and A-lGNPs in different serum solutions at different time points.

Figure S9. ITC analysis of the interaction between FBS-treated A-GNPs and lung surfactants. Curves correspond to the interactions of FBS-treated A-lGNPs with (A) PI and (B) PG and FBS-treated A-sGNPs with (C) PI and (D) PG.

Figure S10. Body weight changes during the treatment by A-sGNPs and A-lGNPs are plotted respectively.

Figure S11. HE staining of the liver and spleen after A-sGNPs and A-lGNPs treatments after 24 h. A-GNPs are marked by yellow arrows. Part of this figure shows magnified views of images adapted from Figure 6A.

Figure S12. Routine blood evaluation of mice at different time points treated by A-sGNPs and A-lGNPs.

Figure S13. The bleeding area ratios in lungs of (A) *P.a* or (B) MDR *P.a* infected mice treated by different agents.

Figure S14. Bacteria colon number within lung of mice after treatment with A-sGNPs, A-lGNPs, antibiotics, or saline for different treatment times are counted and plotted.

Figure S15. Bacteria in the livers of mice with an acute pneumonia caused by *P.a* (left) or MDR *P.a* (right) treated by A-sGNPs, A-lGNPs, antibiotics, or saline at different time points. × represent the dead mice.

Figure S16. Bacteria in the kidneys of mice with an acute pneumonia caused by *P.a* (left) or MDR *P.a* (right) treated by A-sGNPs, A-lGNPs, antibiotics, or saline at different time points. × represent the dead mice.

Figure S17. Gold content in bronchoalveolar lavage fluid at different time points after tail vein injection of A-GNPs in both normal and lung-infected mouse models.

Figure S18. (A) Blood biochemistry evaluation and (B) blood routine tests after the infection by different bacteria at different time points.

Figure S19. The liver and kidney function index of mice infected by different pathogens and treated by different agents.

Table S1. ATT uptake in different cells at different concentrations.

| Concentration | A549-Low | A549-High | HepG2-Low | HepG2-High |
| --- | --- | --- | --- | --- |
| ATT (μg/mL) | 0.481213 | 4.186836 | 0.53304 | 1.180876 |

Table S2. The dissociation constant of A-GNPs and lung surfactants.

| Ka (M^-1^) | A-lGNPs | A-sGNPs |
| --- | --- | --- |
| PI | (2.21±0.468)×10^4^ | (2.22±0.480)×10^4^ |
| PG | (1.17±0.185)×10^4^ | (1.29±0.219)×10^4^ |

Table S3. The dissociation constant of FBS-treated A-GNPs and lung surfactants.

| Ka (M^-1^) | FBS+A-lGNPs | FBS+A-sGNPs |
| --- | --- | --- |
| PI | (1.64±0.389)×10^4^ | (1.76±0.375)×10^4^ |
| PG | (1.30±0.209)×10^4^ | (1.41±0.149)×10^4^ |

Table S4. Antibacterial effect of A-GNPs treated with different types of serums against *P.a*.

| MIC (μg/mL) | A-sGNPs | A-sGNPs  +FBS | A-sGNPs  +MS | A-lGNPs | A-lGNPs  +FBS | A-lGNPs  +MS |
| --- | --- | --- | --- | --- | --- | --- |
| *P.a* | 2.7 | 2.7 | 2.7 | 9.6 | 9.6 | 9.6 |

Table S5. Gold content (ng/mL) in bronchoalveolar lavage fluid at various time points after intravenous injection of A-GNPs.

|  | 2 h | 6 h | 24 h |
| --- | --- | --- | --- |
| A-lGNPs | 359 | 119 | 47 |
| A-sGNPs | 555 | 462 | 256 |

Table S6. Serum tests after the infection by different bacteria at different time points.

|  |  | *P.a* | | | | MDR *P.a* | | | | N |
| --- | --- | --- | --- | --- | --- | --- | --- | --- | --- | --- |
|  |  | 12 h | 24 h | 48 h | 72 h | 12 h | 24 h | 48 h | 72 h |  |
| CRP | Control | 427 | 605 | / | / | 391 | 616 | / | / | 47 |
|  | Antibiotic | 382 | 423 | 305 | 55 | 367 | 395 | 311 | / |  |
|  | A-lGNPs | 395 | 457 | 296 | 80 | 346 | 398 | 225 | 69 |  |
|  | A-sGNPs | 376 | 401 | 244 | 45 | 382 | 387 | 183 | 47 |  |
| PCT | Control | 2116 | 2321 | / | / | 2453 | 2027 | / | / | 298 |
|  | Antibiotic | 1677 | 1823 | 511 | 461 | 1437 | 1403 | 860 | / |  |
|  | A-lGNPs | 1821 | 1747 | 554 | 464 | 1592 | 1521 | 585 | 411 |  |
|  | A-sGNPs | 1422 | 1470 | 496 | 322 | 1288 | 1294 | 464 | 296 |  |
| SAA | Control | 360 | 393 | / | / | 373 | 349 | / | / | 50 |
|  | Antibiotic | 288 | 264 | 73 | 47 | 310 | 284 | 178 | / |  |
|  | A-lGNPs | 305 | 282 | 68 | 49 | 264 | 239 | 93 | 63 |  |
|  | A-sGNPs | 260 | 193 | 58 | 46 | 285 | 245 | 81 | 48 |  |

**References**

38. Feng Y, Chen W, Jia Y, Tian Y, Zhao Y, Long F, Rui Y, Jiang X. N-Heterocyclic molecule-capped gold nanoparticles as effective antibiotics against multi-drug resistant bacteria. *Nanoscale* 2016;8:13223-13227.
